# Canine Neutrophil Extracellular Traps Release Induced by the Apicomplexan Parasite *Neospora caninum In Vitro*

**DOI:** 10.3389/fimmu.2016.00436

**Published:** 2016-10-31

**Authors:** Zhengkai Wei, Carlos Hermosilla, Anja Taubert, Xuexiu He, Xiaocen Wang, Pengtao Gong, Jianhua Li, Zhengtao Yang, Xichen Zhang

**Affiliations:** ^1^College of Veterinary Medicine, Jilin University, Changchun, Jilin, China; ^2^Faculty of Veterinary Medicine, Institute of Parasitology, Justus Liebig University Giessen, Giessen, Germany

**Keywords:** NETs, canine, neutrophils, *Neospora caninum*

## Abstract

Neosporosis is considered as one of the main causes of abortion and severe economic losses in dairy industry. The *Canis* genus serving as one of the confirmed definitive hosts of the apicomplexan parasite *Neospora caninum* (*N. caninum*) plays a critical role in its life cycle. However, the effects of *N. caninum* on its definitive hosts of neutrophils extracellular traps (NETs) formation remain unclear. In the present study, *N. caninum* tachyzoite-induced canine NETs formation was observed by scanning electron microscopy (SEM). Visualization of DNA decorated with H3, neutrophil elastase (NE), and myeloperoxidase (MPO) within *N. caninum* tachyzoite-induced NETs were examined using fluorescence confocal microscopy analyses. Furthermore, the formation of canine NETs was quantified using Sytox Green staining, and the LDH levels in supernatants were examined by an LDH Cytotoxicity Assay^®^ kit. The results clearly showed that NETs-like structures were induced by *N. caninum* tachyzoites, and the major components within these structures induced by *N. caninum* tachyzoite were further confirmed by fluorescence confocal microscopy visualization. These results suggest that *N. caninum* tachyzoites strongly induced NETs formation in canine polymorphonuclear neutrophils (PMN). In functional inhibition assays, the blockings of NADPH oxidase, NE, MPO, SOCE, ERK 1/2, and p38 MAPK signaling pathways significantly inhibited *N. caninum* tachyzoite-induced NETs formation. To our knowledge, this study is the first to report the formation of NETs in canine PMN against *N. caninum* infection.

## Introduction

Neosporosis is caused by the apicomplexan protozoa *Neospora caninum* and is considered as one of the main diseases causing abortion, reproduction disorders, and thus severe economic losses in dairy industry worldwide (1, 2). *N. caninum* is an apicomplexan parasite closely related to *Toxoplasma gondii*, which infects a wide intermediate host range (3, 4). As an important veterinary pathogen, several researches have been focused on *N. caninum* in the past two decades. To date, its confirmed definitive hosts of *N. caninum* is the genus *Canis*, including domestic dogs (*Canis familiaris*) (5), dingoes (*Canis lupus dingo*) (6), and gray wolves (*Canis lupus*) (7). Neosporosis in canines principally results in a neuromuscular disease, i.e., polyradiculoneuritis-myositis, but a variety of less common lesions due to focal necrosis can also occurs in other organs (8, 9). Although several studies on cattle *N. caninum* infections are well reported and analyzed by serologic and/or molecular diagnostic assays, the definitive host–parasite interactions remain not well clarified.

Polymorphonuclear neutrophils (PMN) are one of the most abundant and important leukocyte population in blood, thereby considered as the first-line of defense in the host innate immune system against invasive microorganisms. Neutrophils extracellular traps (NETs) have been recognized as a novel effector mechanism of PMN in many immune processes. NETs are mainly compost of DNA, antibacterial proteins/peptides, and granule proteins, such as histones, neutrophil elastase (NE), myeloperoxidase (MPO), lactoferrin, gelatinase, pentraxin, and cathelicidin, among other molecules (10, 11). During *N. caninum* infection of bovine endothelial cells, PMN adhesion was increased and the expression of adhesion molecules, such as E-selectin, VCAM-1, and ICAM-1, was significantly upregulated, which revealed the critical role of PMN in the host innate immune system (12). However, whether the novel effector mechanism of PMN-NETs involved in the interactions between *N. caninum* and its definitive hosts has not been investigated during the acute infection of *N. caninum*. In this study, the influence of *N. caninum* on canine NETs formation was examined, and the key molecular signaling pathways were further elucidated.

## Materials and Methods

### Parasites

*Neospora caninum* tachyzoites (strain Nc-1) were maintained in VERO cells monolayer at 37°C/5% CO_2_. VERO cells were cultured in RPMI 1640 medium (Hyclone, USA) supplemented with 2% fetal bovine serum (FBS, Biological Industries, Israel) and 1% penicillin/streptomycin (Hyclone, USA). *N. caninum* tachyzoites were collected and harvested from VERO cells (3000 r/min, 10 min, room temperature), then passed through 20, 5, 1-ml syringe and a 27-gage needle in turn, purified by 40% Percoll reagent through centrifugation (3000 r/min, 30 min, room temperature), collected precipitates and washed them twice with RPMI 1640 medium (3000 r/min, 10 min, room temperature).

### Isolation of Canine PMN

Adult healthy canines (*n* = 3) were bleeding by puncture of the femoral vein, and blood was collected in blood collection tubes containing heparin (Jun Nuo, Shandong Chengwu County Medical Products Co., China). The PMN were isolated by using the commercially available Canine PMN isolation kit^®^ (TianJin HaoYang Biological Manufacture Co., China) according to the manufacturer’s instructions. All animal experiments were approved by the NIH Guide for the Care and Use of Laboratory Animals of the Jilin University and in accordance to the current Animal Protection Laws of China.

### Scanning Electron Microscopy

Canine PMN were stimulated with *N. caninum* tachyzoites (ratio 1:1, 90 min, 37°C) on cover glass slides. These cover glass slides were pre-coated with poly-l-lysine (0.1 mg/ml, Sigma-Aldrich) for 12 h and washed three times with distilled water. After incubation, the cells were fixed with 4.0% glutaraldehyde (Merck) for 24 h, washed with PBS and post-fixed in 1.0% osmium tetroxide (Merck). Then, the samples were dehydrated in ascending ethanol concentrations (30, 50, 70, 80, 90, 100%), frozen in tertiary butyl alcohol at −20°C and sputtered with gold. Finally, specimens were examined using a scanning electron microscope (Hitachi S-3400N, Japan).

### Fluorescence Confocal Microscopy Analyses

Canine PMN were stimulated with *N. caninum* tachyzoite (ratio 1:1, 90 min, 37°C) on poly-l-lysine (0.1 mg/ml, Sigma-Aldrich) pre-coated cover glass slides. After incubation, the cells were fixed with 4% (w/v) paraformaldehyde for 20 min at room temperature, washed thrice with PBS, permeabilized with 0.1% Triton X-100 for 15 min and blocked in 3% goat serum/PBS, followed by incubation with antibodies to H3, MPO, and NE at 4°C overnight. Anti-histone antibody (LS-C353149; Life Span BioSciences, Inc, 1:200, dissolved in 3% goat serum), anti-MPO antibody (Orb16003; Biorbyt, 1:200, dissolved in 3% goat serum), and anti-NE antibody (AB68672; Abcam, 1:200, dissolved in 3% goat serum) were used for the detection of H3, MPO, and NE in *N. caninum* tachyzoite-triggered NETs-like extracellular structures. Then, the samples were incubated with the second conjugated antibody (goat anti-rabbit IgG-FITC conjugated, Bioworld Technology Inc) and washed two times with PBS, then stained with 5-μM Sytox Orange (dissolved in PBS, Invitrogen) for 10 min at room temperature. Finally, the specimens were washed two times with PBS, mounted in anti-fading reagents (Beyotime Biotechnology, China), and examined using scanning confocal microscope (Olympus FluoView FV1000).

### Quantitation of NETs

Canine PMN were stimulated with viable *N. caninum* tachyzoites (ratio 1:1, 1:2, 1:3, 1:6, or 1:12, 37°C) for 30, 60, and 90 min, respectively. In parallel settings, prior to stimulation with *N. caninum* tachyzoite (1:6), the canine PMN were pretreated with the following specific inhibitors for 30 min including the NE inhibitor (CMK, 1 mM, Sigma-Aldrich), the NADPH oxidase inhibitor diphenylene iodonium (DPI, 10 μM, Sigma-Aldrich), the MPO inhibitor (ABAH, 100 μM, Calbiochem), the inhibitors of ERK 1/2 (UO126, 50 μM, Sigma-Aldrich) and p38 MAKP (SB202190, 10 μM, Sigma-Aldrich) signaling pathway, and with the store-operated calcium entry (SOCE) inhibitor (2-amindethoxydiphery borate, 100 μM, Sigma-Aldrich) for 15 min. The formation of canine NETs was quantified using 5-μM Sytox Green (Invitrogen). The samples were examined with a fluorometric reader Infiniti M200^®^ (TECAN, Austria) using an excitation wavelength of 488 nm and detecting at 523 nm the emission wavelength.

### Detection of Reactive Oxygen Species

The reactive oxygen species (ROS) production of PMN induced by *N. caninum* tachyzoites (ratio 1:1, 90 min) was determined with 2,7 dichlorofluorescein diacetate (DCFH-DA, Sigma-Aldrich). PMN stimulated with zymosan (1 mg/ml, Sigma) were used as positive controls. The samples were examined with a fluorometric reader using an excitation wavelength of 485 nm and detecting at 530 nm the emission wavelength.

### LDH Detection

The LDH levels in supernatant were examined by the LDH Cytotoxicity Assay kit^®^ (Beyotime Biotechnology, China) according to the manufacturer’s protocols.

### Statistical Analysis

Values were expressed as the means ± SD. Data were analyzed by the GraphPad 5.0 software. Differences among the groups were performed with one-way analysis of variance (ANOVA) with Tukey multiple comparison test. *P* values of <0.05 were considered as significant.

## Results

### *Neospora caninum* Tachyzoites Induced NETs Formation in Canine PMN

**Figure 1:**
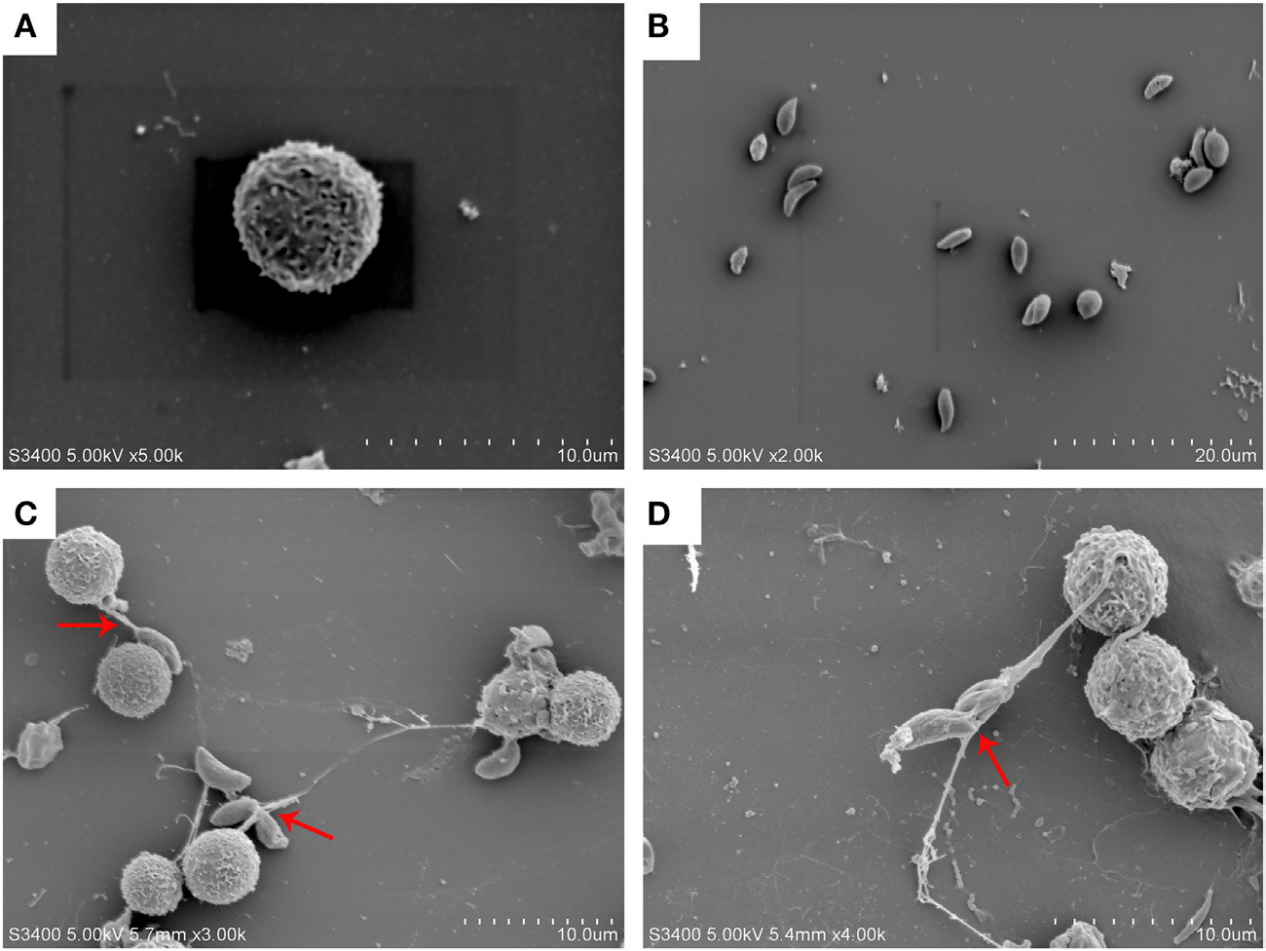
***N. caninum* tachyzoite-induced NETs formation was observed by SEM**. **(A)** PMN. **(B)** *N. caninum* tachyzoites. **(C,D)** NETs were induced by *N. caninum* tachyzoite, and *N. caninum* tachyzoites were trapped in these thicker and thinner network structures.

**Figure 2:**
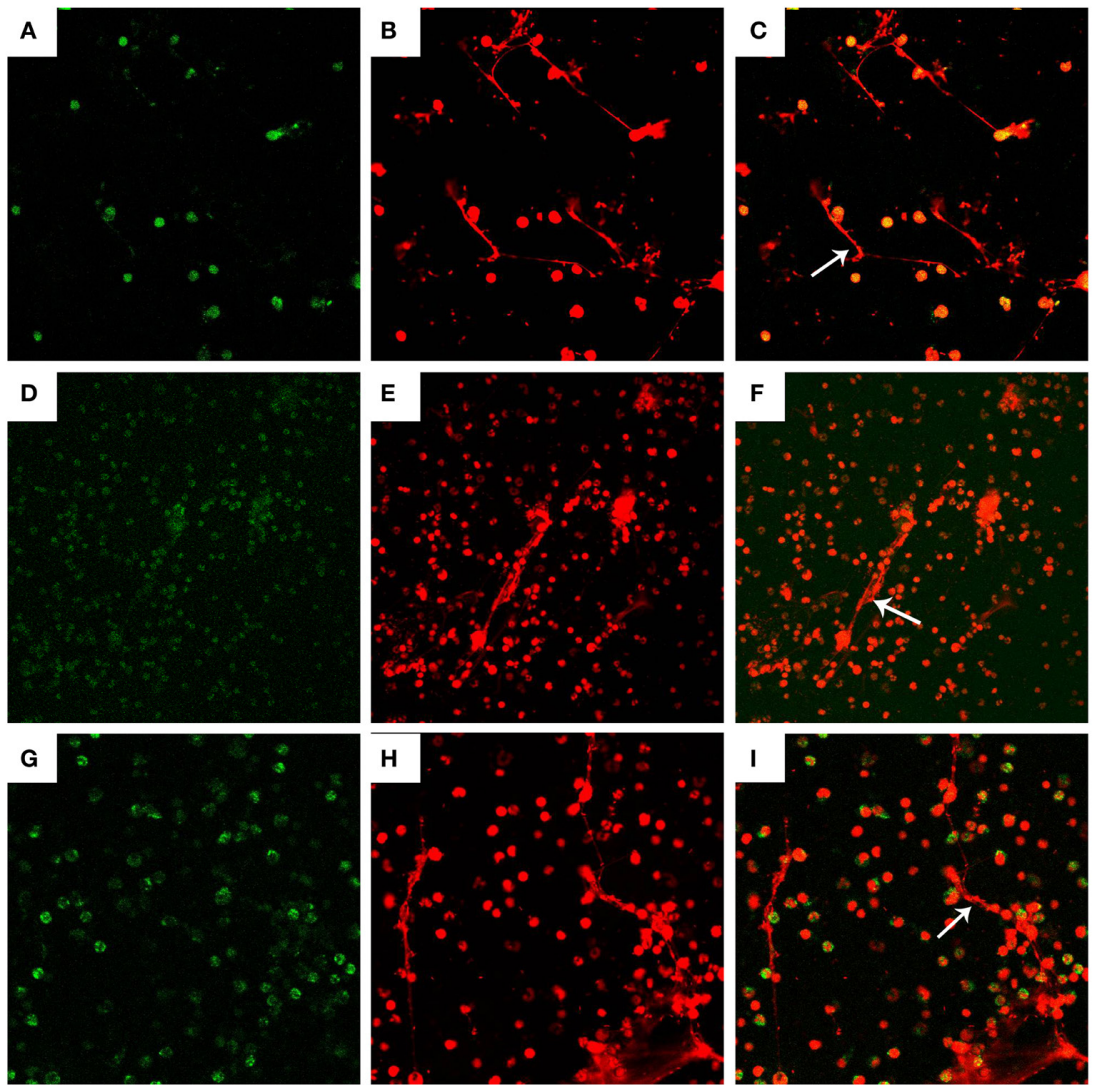
**Visualization of DNA decorated with histones (H3), neutrophil elastase (NE), and myeloperoxidase (MPO) in *N. caninum* tachyzoite-induced NETs structures**. PMN were stimulated with *Neospora caninum* (ratio: 1:1, 90 min). DNA decorated with of H3, NE, and MPO within NETs were detected using a scanning confocal microscope. **(A)** Histone (Green). **(D)** MPO (Green). **(G)** NE (Green). **(B,E,H)** DNA (Red). **(C,F,I)** Respective merge of DNA decorated with H3, NE, and MPO.

*Neospora caninum* tachyzoites strongly induced NETs formation in canine PMN and was confirmed by scanning electron microscopy (SEM) analyses (Figure 1). NETs released from canine PMN were observed in Figure 1, and *N. caninum* tachyzoites were captured in these thicker and thinner network extracellular structures. Furthermore, *N. caninum* tachyzoite-induced NETs in canine PMN were demonstrated by fluorescence confocal microscopy analyses (Figures 2B,E,H). These results confirmed that *N. caninum* tachyzoite surely induced NETs in canine PMN as an additional anti-parasitic effector mechanism.

### Visualization of DNA Decorated with Histones (H3), Neutrophil Elastase, and Myeloperoxidase in *N. caninum* Tachyzoite-Induced NETs Structures

Fluorescence confocal microscopy analyses revealed that NETs formation can be induced by *N. caninum* tachyzoites, and the nature of these structures was mainly composed by DNA (Figures 2B,E,H). Besides DNA backbone structure of *N. caninum*-induced NETs, H3 (Figure 2A), MPO (Figure 2D), and NE (Figure 2G) were also observed corroborating the colocalization of these molecules and DNA of these NETs. Merge image of DNA decorated with H3, MPO and NE was showed in Figures 2C,F and I. Taken together, these results confirm the main classical characteristics of NETs induced by *N. caninum* tachyzoites in canine PMN.

### Quantitation of NETs

**Figure 3:**
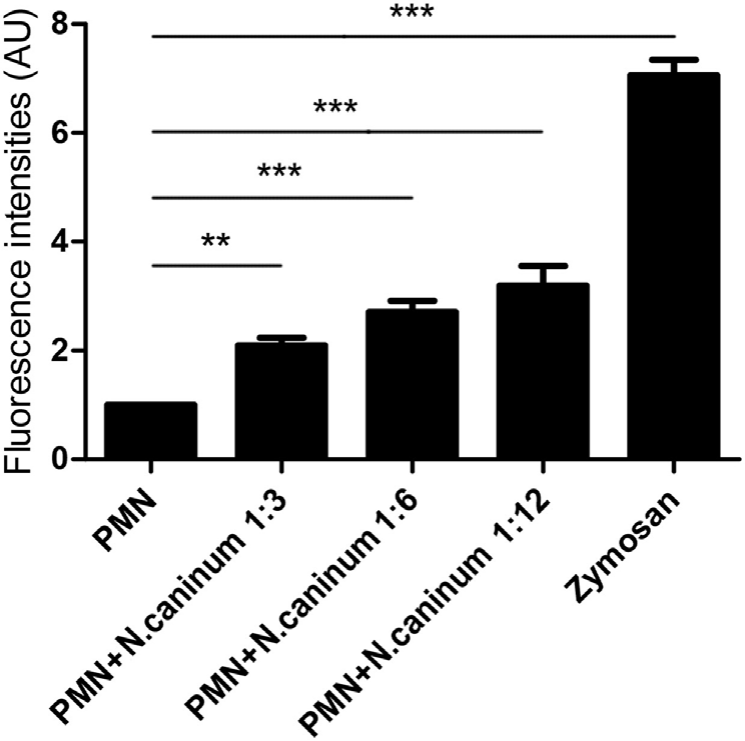
***N. caninum* tachyzoite-induced NETs formation was in a dose-dependent manner**. Canine PMN were stimulated with *Neospora caninum* tachyzoite (PMN: tachyzoite = 1:3, 1:6, and 1:12) for 60 min. The formation of *N. caninum* tachyzoite-induced NETs was examined with a fluorometric reader using an excitation wavelength of 488 nm and detecting at 523 nm. Values are presented as mean ± SD (*n* = 5). *P* values of <0.05 were considered significant (**P < 0.01 and ***P < 0.001).

**Figure 4:**
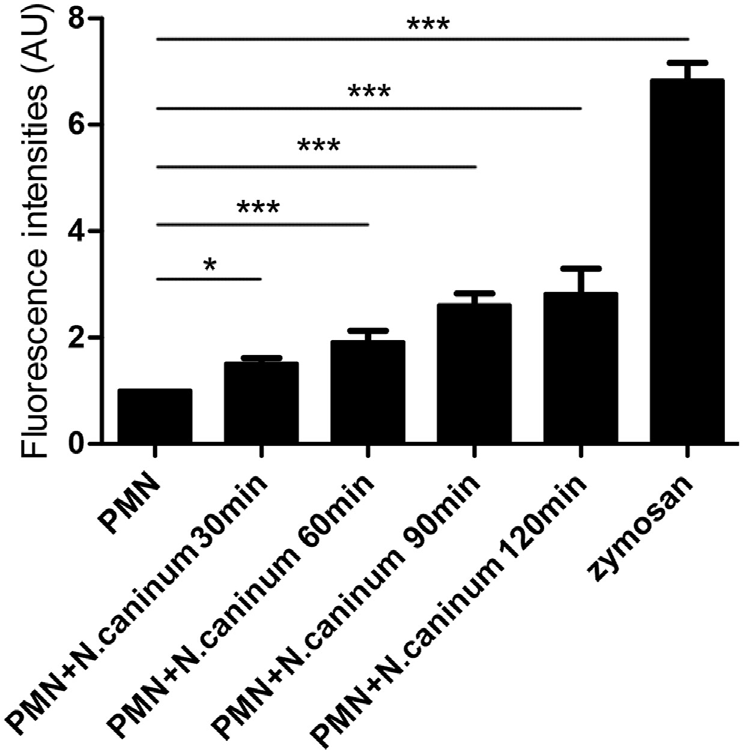
**Kinetics of *N. caninum* tachyzoite-induced NETs formation**. PMN were stimulated with *Neospora caninum* tachyzoite (PMN: tachyzoite = 1:2) for 30, 60, 90, and 120 min. The formation of *N. caninum* tachyzoite-induced NETs was examined with a fluorometric reader using an excitation wavelength of 488 nm and detecting at 523 nm. Values are presented as mean ± SD (*n* = 5). *P* values of <0.05 were considered significant (*P < 0.05 and ***P < 0.001).

Canine NETs formation was quantified using Sytox Green. As shown in Figures 3 and 4, *N. caninum* tachyzoites significantly induced increasing quantitation of NETs when compared to negative controls but lower levels than zymosan (positive controls). These results revealed that the formation of NETs induced by *N. caninum* tachyzoite was a dose- and time-dependent process.

### NADPH Oxidase, NE, and MPO Are Involved in *N. caninum* Tachyzoite-Induced NETs Formation

**Figure 5:**
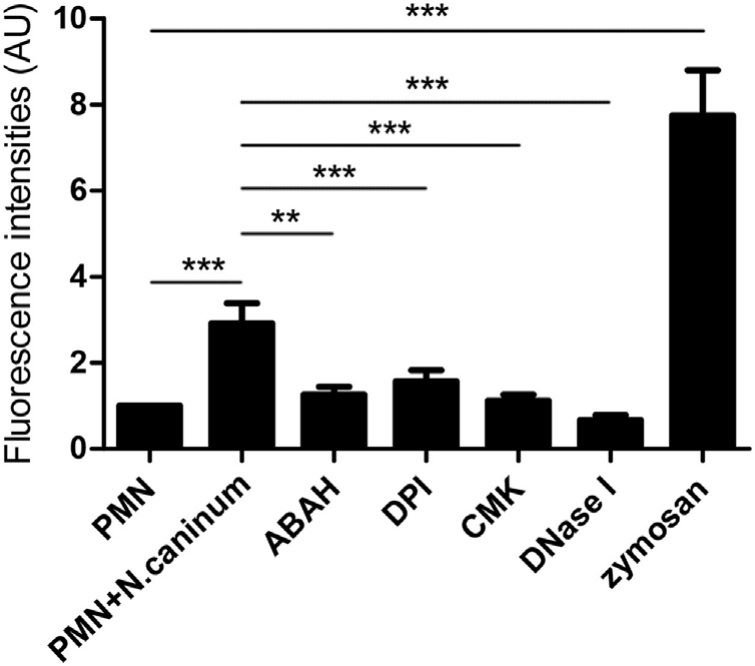
**Inhibition assay of *N. caninum* tachyzoite-induced NETs in canine PMN**. Prior to stimulation with *N. caninum* tachyzoite, the PMN were pretreated with the following inhibitors of NADPH oxidase, NE and, MPO. Zymosan was used as positive controls. The formation of canine NETs was quantified using Sytox Green (Invitrogen). The samples were examined with a fluorometric reader using an excitation wavelength of 488 nm and detecting at 523 nm. Values are presented as mean ± SD (*n* = 5). *P* values of <0.05 were considered significant (**P < 0.01 and ***P < 0.001).

**Figure 6:**
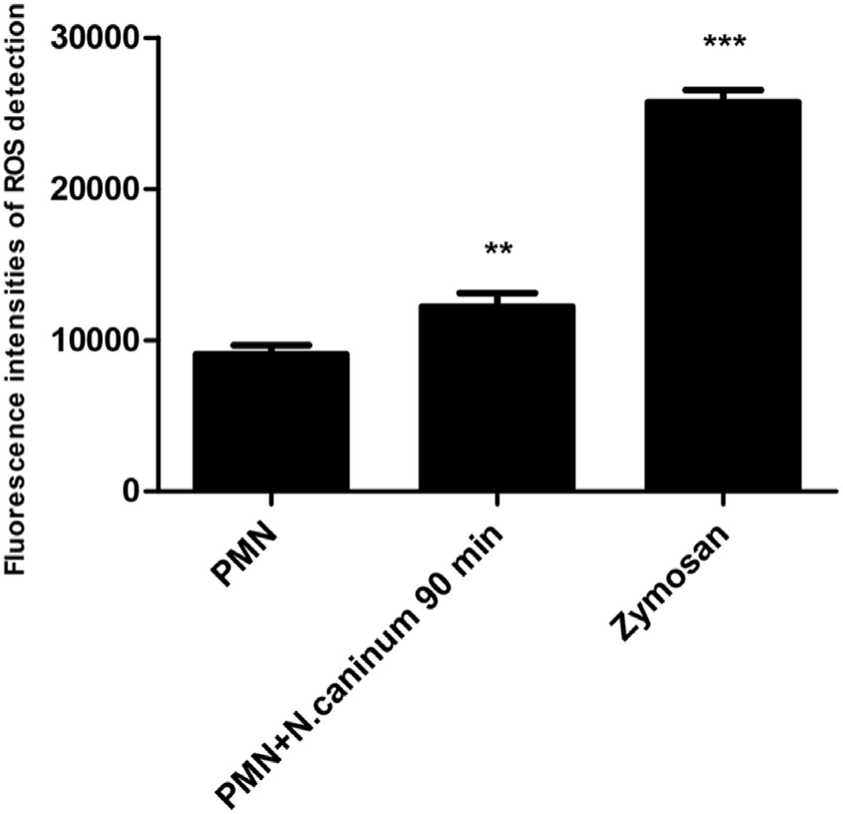
**ROS production of canine PMN induced by *N. caninum* tachyzoite**. The ROS production of PMN induced by *N. caninum* tachyzoite (ratio 1:1, 90 min) was determined with 2,7 dichlorofluorescein diacetate (DCFH-DA, Sigma). PMN stimulated with zymosan (1 mg/ml, Sigma) were used as positive controls. Values are presented as mean ± SD (*n* = 5). *P* values of <0.05 were considered significant (**P < 0.01 and ***P < 0.001).

To investigate the role of NADPH oxidase, NE, and MPO of whether to be involved in *N. caninum* tachyzoite-induced NETs, inhibitors of NADPH oxidase, NE, and MPO were here used. As shown in Figure 5, DPI, ABAH, CMK, and DNase I treatments significantly inhibited *N. caninum* tachyzoite-induced NETs. In addition, *N. caninum* tachyzoites significantly increased ROS production when compared to negative controls (Figure 6). These results suggest that NADPH oxidase, NE, and MPO are involved in *N. caninum* tachyzoite-induced NETs formation. Moreover, the DNase I treatment resulted in significant reduction of *N. caninum*-induced NET formation, thereby confirming that the primary nature of these NETs was a DNA backbone.

### *Neospora caninum* Tachyzoite-Induced NETs Formation Is an ERK 1/2-, p38 MAPK-, and SOCE-Dependent Process

**Figure 7:**
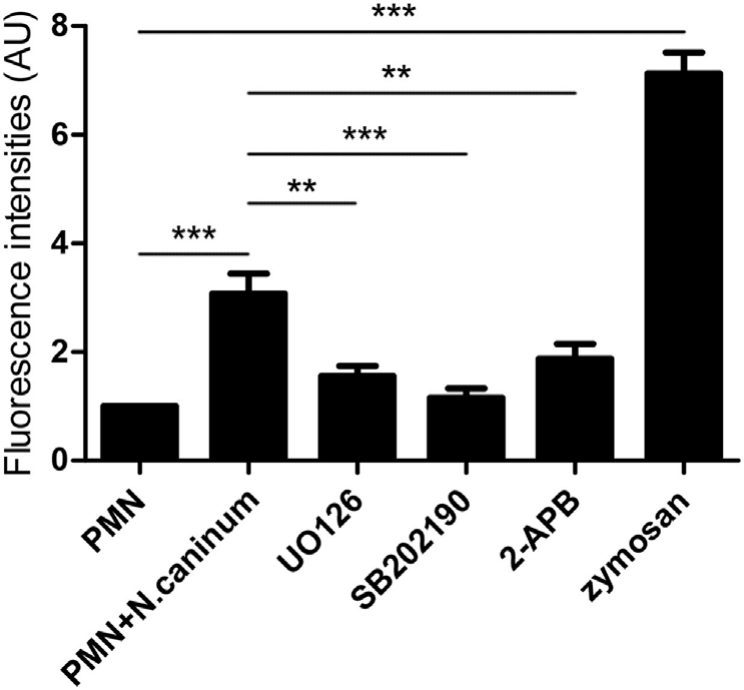
**Effects of SOCE inhibitor, ERK, and P38 signaling pathway inhibitors on *N. caninum* tachyzoite-induced NETs in canine PMN**. Prior to stimulation with *N. caninum* tachyzoite, the PMN were pretreated with the following inhibitors of SOCE, ERK, and P38 signaling pathway. Zymosan was used as positive controls. The formation of canine NETs was quantified using Sytox Green (Invitrogen). The samples were examined with a fluorometric reader using an excitation wavelength of 488 nm and detecting at 523 nm. Values are presented as mean ± SD (*n* = 5). *P* values of <0.05 were considered significant (**P < 0.01 and ***P < 0.001).

In order to investigate in more detail, molecular signaling pathways of *N. caninum*-triggered NET formation, inhibition assays were performed. For this purpose, inhibitors of the SOCE, ERK1/2, and p38 MAPK signaling pathways were used to analyze the critical role of Ca^2+^ and these two kinases-dependent signaling pathways. As shown in Figure 7, 2-APB, UO126, and SB202190 significantly inhibited *N. caninum* tachyzoite-induced NETs. These results indicated that *N. caninum* tachyzoite-induced NETs formation was an ERK 1/2 and p38 MAPK signaling pathway- and SOCE-dependent process.

### *N. caninum* Tachyzoite-Induced NETs Formation Is Independent of LDH Activity

**Figure 8:**
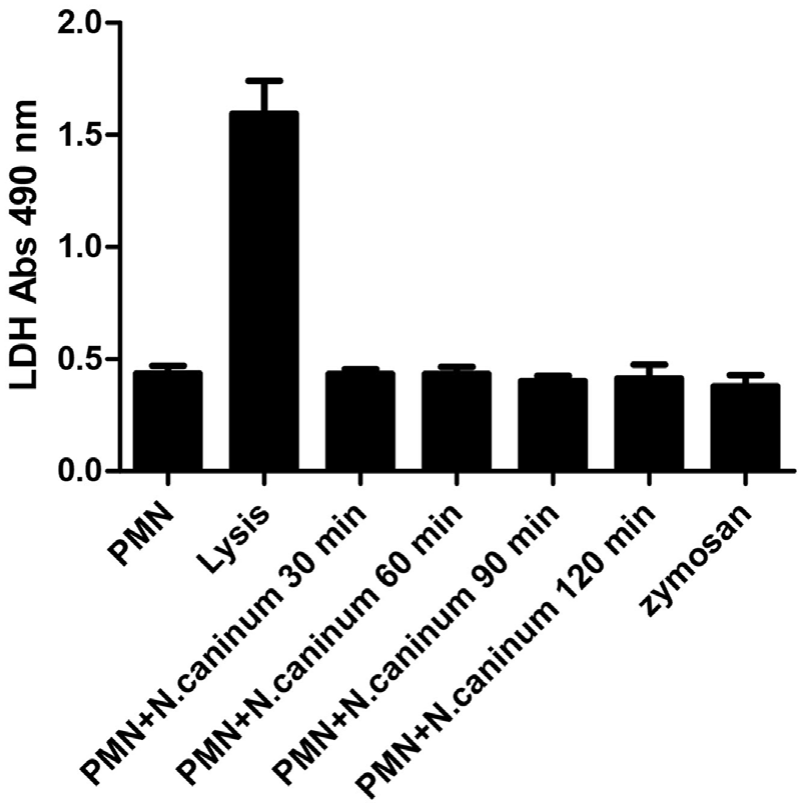
**LDH activity in *N. caninum* tachyzoite-induced NETs formation**. Canine PMN were stimulated with *N. caninum* tachyzoite (ratio 1:1) for 30, 60, 90, and 120 min. The LDH levels in supernatant were examined by an LDH Cytotoxicity Assay kit according to the manufacturer’s protocols. Values are presented as mean ± SD (*n* = 5). *P* values of <0.05 were considered significant (***P < 0.001).

To account for the novel form of cell death program-NETosis, we tested LDH activity in the progress of *N. caninum* tachyzoite-induced NETs formation. As shown in Figure 8, LDH activities in the supernatant were markedly induced by lysis buffer, but there were no significant changes in the progress of *N. caninum* tachyzoite-induced NETs formation.

## Discussion

As the first-line of defense within the host innate immune system, PMN are endowed with powerful effector mechanisms, such as phagocytosis and degranulation, to resist and kill microbes (13). In 2004, another novel effector mechanism of PMN was first described and named “NETs” (10). This novel effector mechanism has been considered as a physical barrier to trap microbes and to avoid their dissemination, and even kill these entrapped microbes. It has been demonstrated that several stimuli, including bacteria, fungi, viruses, and crystal salts can induce the activation of this novel mechanism-NETs in activated PMN (14–16). While most researches concentrated on the effects of NETs on bacteria, fungi, and viral pathogens, increasing evidence on NETs formation triggered by parasites was reported recently, such as *T. gondii, Eimeria bovis*, and *Leishmania donovani* (17–19). However, the effects of *N. caninum* on NETs formation have not been investigated. SEM analysis revealed that NETs-like structures in canine PMN was induced by *N. caninum* tachyzoites *in vitro*, and *N. caninum* tachyzoites were captured by these thicker and thinner network structures. Furthermore, these NETs-like structures induced by *N. caninum* tachyzoites were demonstrated by fluorescence confocal microscopy analysis. Quantitative assays also revealed that *N. caninum* tachyzoite-induced NETs formation was a time- and dose- dependent process. These results confirmed that *N. caninum* tachyzoites can clearly induce NETs *in vitro*, which is in accordance to NETs-related data for *T. gondii, E. bovis*, and *L. donovani* (17–19). However, we do not know the effects of NETs on *N. caninum* infection in bovine, and whether neutrophils would also be able to recognize and attack *N. caninum* by NETs formation *in vivo* need to be further investigated.

In response to pathogens infection, the process of NETs formation has been accompanied by the concentration of several antibacterial proteins and granule proteins, including histones, MPO, NE, and cathelicidin at the site of infection (20–22). In this study, the decoration of DNA with H3, MPO, and NE in *N. caninum*-triggered NETs were demonstrated. These colocalization results clearly proved the nature of these NET structures after the exposure of canine PMN to vital tachyzoites. Furthermore, inhibitors of NE and MPO significantly inhibited *N. caninum* tachyzoite-induced NETs formation, which suggest the critical role of NE and MPO in *N. caninum* tachyzoite-induced NETs. These proteins were also previously observed as key molecules in *T. gondii-, Besnoitia besnoiti*-, and *E. bovis*-triggered ETs (17, 23, 24). Previous studies showed that NETs formation was a NADPH oxidase-dependent process, which resulted in the production of ROS (25). Thus, DPI was used to investigate the role of NADPH oxidase in *N. caninum* tachyzoite-induced NETs. As a result, pretreatment with the NADPH oxidase inhibitor significantly decreased *N. caninum* tachyzoite-induced NETs formation, and *N. caninum* tachyzoite significantly increased ROS production. Moreover, ROS production has been proved to be dependent on SOCE (26), so we next explored the role of SOCE in *N. caninum* tachyzoite-induced NETs formation. The results showed that *N. caninum* tachyzoite-induced NETs formation was significantly decreased by the SOCE inhibitor 2-APB. There is another report showing that SOCE is regulated *via* ERK 1/2 phosphorylation and *via* a ROS production-dependent activation of the Raf–MEK–ERK pathway, which has also been proved to be required for NETs formation induced by parasites (23, 27, 28). Then, we analyzed the critical role of Ca^2+^ influx and the role of ERK 1/2 and p38 signaling pathway in *N. caninum* tachyzoite-induced NETs. Our results show that 2-APB, UO126, and SB202190 significantly inhibited *N. caninum* tachyzoite-induced NETs formation thereby proving the potential role of these molecules in *N. caninum*-mediated NETosis. As reported for several pathogens, *N. caninum* tachyzoite-induced NETs formation may be also a NADPH oxidase-, NE-, MPO-, SOCE-, ERK 1/2-, and p38 MAPK-dependent process (23, 28). In addition, we detected LDH activity in the process of *N. caninum* tachyzoite-induced NET formation. These results showed that no significant necrosis occurred during *N. caninum* tachyzoite-induced NETs formation when compared to positive controls, which was in accordance with the characteristics of typical NETosis induction as previously demonstrated (10, 15, 29).

Taken together, *N. caninum* tachyzoites are strong inducers of canine NETs, which suggested a critical role of NETs in the early host innate immune response against tachyzoites. Whether other *N. caninum* stages, such as sporozoites or bradyzoites, might be capable to induce NETosis needs further investigations. Furthermore, several molecular mechanisms have been proved to be involved in *N. caninum* tachyzoite-induced NETs formation. However, the role of NETs in *N. caninum* infection *in vivo* during acute neosporosis calls for more investigations.

## Author Contributions

ZY, CH, AT, and XZ designed the project and experiments. ZW, XH, and XW carried out most of the experiments. ZW and CH wrote the manuscript. ZW, PG, and JL carried out statistical analysis and prepared figures. ZY and XZ co-corresponded this paper. All authors reviewed the manuscript.

## Conflict of Interest Statement

The authors declare that the research was conducted in the absence of any commercial or financial relationships that could be construed as a potential conflict of interest.
